# Relative improvement in language vs. motor functions with reperfusion therapies for large vessel occlusion

**DOI:** 10.1038/s41598-025-90871-x

**Published:** 2025-02-24

**Authors:** Zaka Ahmed, Vivek Yedavalli, Wladimir Sarmiento Gonzalez, Argye E. Hillis

**Affiliations:** 1 Department of Neurology, Johns Hopkins School of Medicine, Baltimore, USA; 2 Department of Radiology, Johns Hopkins School of Medicine, Baltimore, USA; 3 Departments of Physical Medicine & Rehabilitation, Johns Hopkins School of Medicine, Baltimore, USA; 4 Department of Neurology, Cerebrovascular Division, Sheikh Khalifa Endowed Chair of Excellence in Stroke Detection and Treatment, Johns Hopkins University School of Medicine, Phipps 446, 600 N. Wolfe Street, Baltimore, MD 21287 USA

**Keywords:** Acute ischemic stroke, NIH stroke scale, Aphasia, Hemiparesis, Endovascular treatment, Thrombolysis, Neurology, Signs and symptoms

## Abstract

When weighing potential risks versus benefits of reperfusion therapy, the functions likely to recover if blood flow can be restored should be considered. Because deep and motor areas often infarct relatively early in acute stroke, we hypothesized that reperfusion therapies improve predominantly cortical functions more than motor function. In this retrospective review of a prospectively collected database of patients with acute stroke due to large vessel occlusion, we evaluated percent improvement (mean change in score/maximum score) for different items of the National Institutes of Health Score Scale with and without endovascular thrombectomy (EVT), and/or intravenous thrombolysis. In total, 290 patients met the inclusion criteria. For all outcome measures there were significant effects of treatment group (*p* < 0.0001 for all), with the greatest change in the EVT + thrombolysis group, then EVT only group, followed by thrombolysis only, followed by no intervention. Differences between EVT + thrombolysis and EVT only were not significant (*p* = 0.30 to 0.79 across outcomes). For patients with aphasia and/or right sided weakness before treatment, the percent change in language was significantly greater than the percent change in weakness (29.8% vs. 12.7%; t(93) = 5.3;*p* < 0.0001). Greater percent improvement in language was observed in all treatment groups (*p* = 0.0003 to 0.03 across treatment groups). After acute ischemic stroke due to LVO, improvements occur in all neurological functions with intervention. However, gains in language are even greater than gains in motor function in the same patients. Few patients had neglect before treatment, but of those who did, the majority improved, and most (92.8%) with EVT.

**Supplementary Information:**

The online version contains supplementary material available at 10.1038/s41598-025-90871-x.

## Introduction

Treatment of acute ischemic stroke is focused on improving perfusion, both to salvage tissue and to improve function. Endovascular thrombectomy (EVT) is a minimally invasive procedure that includes a variety of interventions to remove the clot that is causing ischemia, using catheters inserted into the arteries. Intravenous thrombolysis aims to dissolve the clot causing ischemia by giving medication (e.g. tissue plasminogen activator, or tPA) intravenously. Both EVT and thrombolysis are effective treatments for acute ischemic stroke secondary to large vessel occlusion (LVO). However, they also carry some risks. When clinicians, patients, and their caregivers weigh the potential risks versus benefits of reperfusion therapy, they should consider what functions are likely to recover if blood flow can be restored. Little information is currently available to allow clinicians to know what functions are likely to improve with reperfusion. Because deep and motor areas of the brain, including caudate, putamen, insular ribbon, middle frontal gyrus, frontal lobe subcortical white matter, precentral gyrus, and frontal lobe paracentral lobule are particularly vulnerable to hypoperfusion and infarct relatively early in acute stroke^1^, we hypothesized that reperfusion therapies are more likely to improve language function and neglect (which depend on cortical areas), more than motor function. That is, successful restoration of blood flow might be more likely to salvage areas that are not already ischemic, including areas critical for language^2–4^ and spatial attention^5–7^, such as left inferior frontal gyrus, temporal cortex, and inferior parietal cortex. Although language and spatial attention clearly depend on complex cortical-subcortical networks, our previous studies have shown that hypoperfusion of cortical regions (or thalamus) is necessary to cause frank aphasia and neglect in acute stroke, and reperfusion of these cortical regions results in recovery^5,8–11^. The goal of our study is to evaluate the percent improvement (mean change in score/maximum score) for different items of the National Institutes of Health Score Scale (NIHSS) with and without EVT, and/or thrombolysis in series of patients who had LVO, pretreatment CT angiogram (CTA) to confirm LVO, and NIHSS for evaluation of acute ischemic stroke (a convenience sample from three hospitals).

## Methods

### Participants

In this IRB approved retrospective study of our prospective collected database, patients with AIS caused by an LVO (defined as distal internal artery, M1, or proximal M2 middle cerebral artery segments) on CTA from 2017 to 2022 from three centers within our larger hospital enterprise. Demographics and interventions received are described in the Results section. Due to the retrospective nature of the study, The Johns Hopkins Institutional Review Board waived the need of obtaining informed consent.

### Behavioral scoring

On the NIHSS, language is evaluated with a 3-point scale (0 = no aphasia, 1 = mild-moderate aphasia, and 2 = severe aphasia). There are additional items that require language: 1b. Level of consciousness questions (0–2 points) and 1c. Level of consciousness commands (0–2 points), such that a Total Language score ranges from 0 to 6). Motor function includes 4 points strength of each arm, 4 points strength of each leg, and 2 points for facial strength (1 point for unilateral facial weakness). Since our participants all had unilateral MCA or ICA occlusions, we assumed their stroke-induced motor dysfunction would be unilateral, for a total range = 0–9). Neglect (11. Extinction and inattention) is assessed with a single item of 0–2 points. To evaluate percent change in each function, for each patient we divided the change in score from pre- to post-treatment, by the maximum possible score for that function, and multiplied by 100. For example, if a patient improved from severe aphasia to mild-moderate aphasia, the percent change would be ½ x 100 = 50% for the language item; if the same person improved from complete right hemiplegia of the face, arm, and leg, the percent change in motor function would be 9/9 × 100, or 100%. All participants were scored for each of the functions of interest. All participants were administered the NIHSS at admission and discharge from the stroke service (a requirement of our stroke center).

### Statistical analysis

We first evaluated percent change (change in score/maximum score) in language, total language (language item + orientation questions and commands), motor (strength in arms and legs and face) across treatment groups (thrombolysis only, EVT only, EVT plus thrombolysis, and no reperfusion therapy) using ANOVA. For all further analyses, we combined EVT only and EVT plus thrombolysis, because there were only small differences between these two groups in percent change in the items of interest (see Table 1 for exact numbers for each group). We then used paired t-tests to evaluate differences in percent change in total motor function versus the language item (and total language score) for participants who were aphasic (1 or more points on the language item) and/or had right sided weakness of the arm, leg, or face (1 or more points). We also used paired t-tests to evaluate differences in percent change in total motor function versus neglect/extinction for participants who were had neglect/extinction (1 or more points on this item) and/or had weakness of the left arm, leg, or face (1 or more points). Finally, we carried out Fisher’s exact tests to evaluate the association between improvement (dichotomous value as 0 and > 0 points change) and each treatment. P value of = < 0.05 was considered significant.

**Table 1:** Percent change in each function and total change in NIHSS score for all patients.

	Mean (SD) change				
	Language item	Total language	Neglect	Motor	NIHSS score
Thrombolysis only (*N* = 37)	14.4%	7.8%	9.5%	5.8%	2.84
	(30.0%)	(17.9%)	(23.1%)	(15.3%)	(0.70)
EVT + thrombolysis (*N* = 21)	31.7%	27.0%	16.7%	27.1%	9.86
	(37.2%)	(33.1%)	(28.9%)	(20.1%)	(1.39)
EVT only (*N* = 18)	22.2%	20.4%	19.4%	20.8%	9.00
	(36.2%)	(28.1%)	(34.9%)	(16.6%)	(1.54)
No intervention (*N* = 214)	4.7%	4.1%	1.9%	2.5%	1.40
	(18.6%)	(15.6%)	(10.7%)	(9.1%)	(0.25)
Total (*N* = 290)	9.0%	7.3%	5.0%	5.9%	2.67
	(24.6%)	(19.7%)	(17.7%)	(13.8%)	
ANOVA:					
*F*	15.65	12.91	11.80	38.76	40.54
p-value	< 0.0001	< 0.0001	< 0.0001	< 0.0001	< 0.0001

## Results

A total of 290 patients with LVO were included in the analyses. Mean age was 61.8 (SD 14.0; range 18–97); 139 (47.9%) were female. MRI confirmed infarct was in the left hemisphere in 150 (51.7%) and right hemisphere in 140 (48.3%).

Of the 290 patients, 37 (12.8%) received thrombolysis only; 21 (7.2%) underwent EVT and thrombolysis; 18 (6.2%) underwent EVT only, and 214 (73.8%) received neither treatment. In this study, the thrombolysis used was intravenous tPA for all patients. Patients who received neither treatment had one or more contra-indications (most commonly delayed arrival). The mean NIHSS score was 4.7 (SD 4.4) in the thrombolysis group, 11.8 (SD 7.1) in the EVT plus thrombolysis group, 10.8 (SD 7.0) in the EVT only group, and 2.7 (SD 4.4) in the group who received neither intervention.

For the entire population (*n* = 290), there were significant differences between treatment groups for all outcome measures (Table 1). For all outcome measures (percent change in language, total language, motor, and neglect) there were significant effects of treatment group (*p* < 0.0001 for all), with the greatest change in the EVT + thrombolysis only group, then EVT only group, followed by thrombolysis only, followed by no intervention. For remaining analyses, we combine EVT only and EVT + thrombolysis, since the differences were small and non-significant (by t-test) for all outcomes. However, the results for EVT only and for EVT + thrombolysis groups are given in the supplement (Table 1S). For both groups, the trends were the same as for the combined EVT (with or without thrombolysis), but differences in percent gains were not statistically significant, given the small numbers in each group. Likewise, hereafter, we also report results for language item alone, rather than total language, as there was generally less change in orientation and simple commands, but no significant difference between the percent improved in the two outcomes with thrombolysis (*p* = 0.80) or EVT (*p* = 0.70) (see Table 1).

The most important comparison in improvement is among those patients who had deficits at baseline. Therefore, we compared percent change in each function for the subset who had deficits at baseline (Table 2). For patients with aphasia and/or right sided weakness (*n* = 94), the percent change in language was greater than the percent change in weakness (29.8 vs. 12.4; t = 5.3; df93; *p* < 0.0001; see Table 2 for 95% confidence intervals) for the same patients. For this subset of 94 patients who had deficits at baseline, the greater improvement in language than motor function was observed for all treatment groups (Table 2). For those who received thrombolysis alone (*n* = 16) the mean difference was 35.4% (SD 35.4) versus 6.6% (SD 14.3) (t = 3.1; df15; *p* = 0.008). For those who received EVT (with or without thrombolysis) (*n* = 23),the difference for percent change in language vs. weakness was 46.4% (SD 37.3) versus 28.1% (SD19.0) (t = 2.3; df22; *p* = 0.03). For patients who received neither EVT nor IV thrombolysis (the largest group, *n* = 55), the difference was 21.2% (SD 29.7) versus 7.5% (SD 15.7) (t = 3.9; df54; *p* = 0.0003). Relatively few patients had neglect at baseline, and there was no significant difference in percentage improvement in neglect versus weakness, in those with neglect and/or weakness pretreatment, with either intervention. However, among the few patients with neglect *and* weakness pretreatment, there was greater percent improvement in neglect compared to motor function (66.7 ± 28.9 versus − 1.7% ±3.0; t = 4.3; df2; *p* = 0.0498).

**Table 2:** Percent change in each function among those with pre-treatment deficits.

	Mean	Standard deviation	95% confidence interval
Combined groups (*N* = 94)			
Percent change in language	29.8	34.0	22.8–36.8
Percent change in motor	12.4	18.5	8.6–16.1
Thrombolysis only (*N* = 16)			
Percent change in language	35.4	35.4	16.5–54.3
Percent change in motor	6.6	14.3	-1.1–14.2
EVT (with or without thrombolysis) (*N* = 23)			
Percent change in language	46.4	37.3	30.3–62.5
Percent change in motor	28.1	19.0	20–36.3
No intervention (*N* = 55)			
Percent change in language	21.2	29.7	13.2–29.2
Percent change in motor	7.5	15.7	3.2–11.7

Only 26 patients had neglect/extinction pre-treatment; 6/8 (75%) who received thrombolysis showed some improvement in neglect; 13/14 (92.9%) who received EVT showed some improvement with treatment; and 7/10 (70%) showed some improvement with neither intervention (ns by Fisher’s exact).

**Fig. 1:**
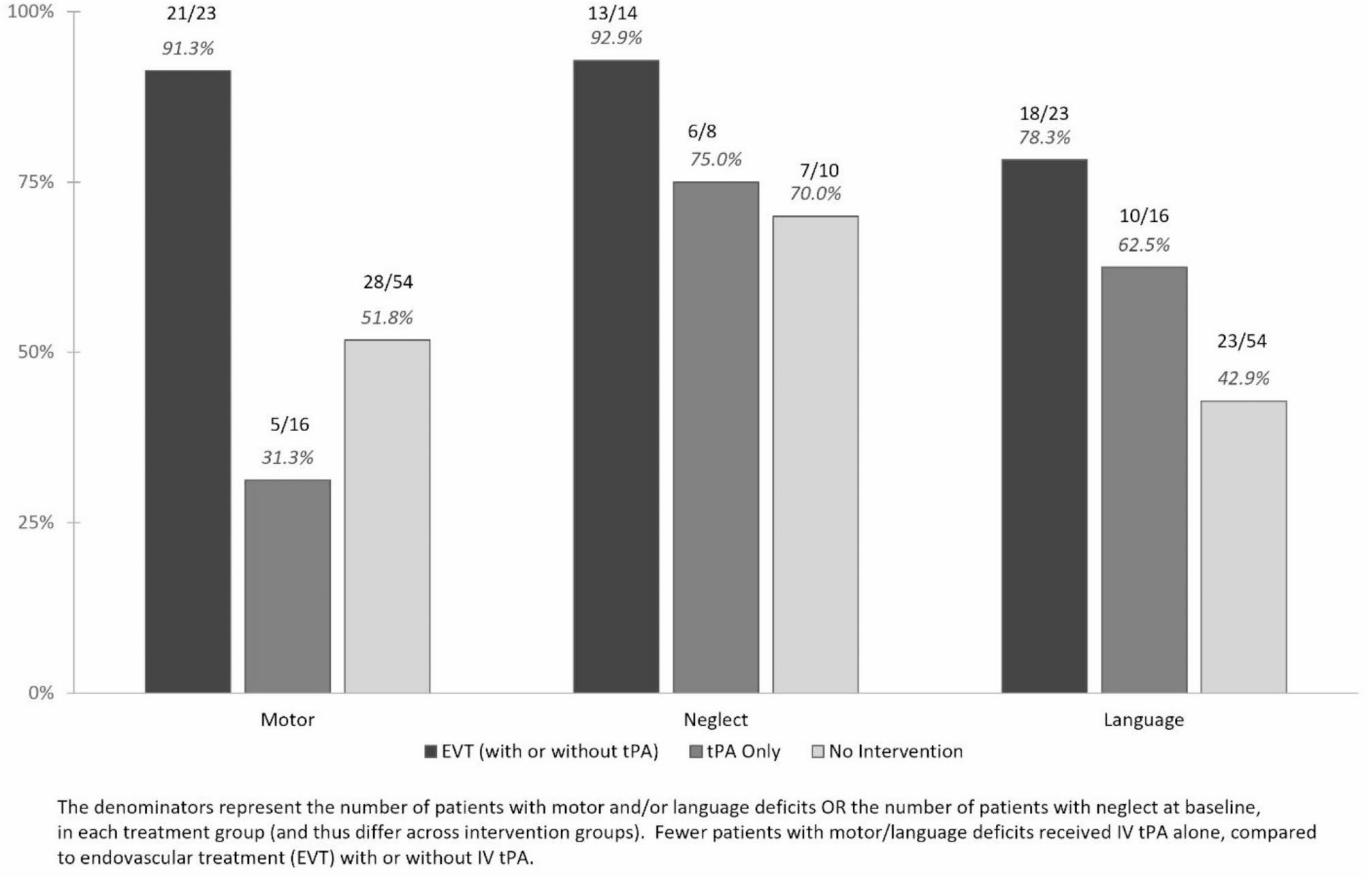
Percentage of patients who improved in each function with each treatment (of those with pre-treatment deficits).

The association between change in each outcome measure (as a dichotomous value) and treatment group was significant by Fisher’s exact both language and strength. Figure 1 shows the percentage of patients who showed any improvement on language, weakness, or neglect for those who had deficits at baseline. Interestingly, of those with aphasia and/or right sided weakness at baseline, 62.5% improved in language with thrombolysis, 78.3% improved in language with EVT, and fewer than half (42.9%) improved with neither intervention. For all functions, improvements were greatest with EVT, then thrombolysis, then no treatment. The difference between treatment groups was significant by Fishers Exact for language (*p* = 0.01) and motor function (*p* < 0.0001). A greater percentage of patients showed some improvement in strength than language without either intervention, while a greater percentage of patients showed some improvement in language than strength with thrombolysis (Fig. 1). However, there were no significant differences in percentage of patients who made any improvement in language or any improvement in strength, of those who were aphasia and/or weak pre-treatment, for any of the intervention groups (by Fisher’s exact).

## Discussion

Consistent with our hypothesis, the predominantly cortical function of language improved more (measured as percent change in score) than strength in the same patients, with EVT and/or thrombolysis. Unsurprisingly, EVT had an enormous effect on language, motor, and spatial attention functions; and thrombolysis had a significant, but smaller effect. However, among those with no intervention aimed to restore blood flow, a higher percentage of patients with deficits at baseline improved in strength than in language (or spatial attention). Therefore, it is unlikely that results can be accounted for by a more sensitive assessment item for language than for motor strength. Rather, results may reflect that reperfusion achieved with EVT or thrombolysis (or both), affects cortical regions that affect language more than strength. While reperfusion of the motor strip would also improve motor function (as reflected in strong effects of treatment on motor function), early infarct of subcortical tissues may limit the percent improvement in strength.

Our results are important for weighing the risks and benefits of interventions and for counseling regarding prognosis. Patients and families should be advised that deficits such as aphasia might improve more than hemiplegia with intervention, although patients are likely to show at least some improvement in strength with or without treatment (albeit more with treatment).

This information may also have implications for selecting outcome measures for interventions to restore blood flow in acute stroke. Currently, the most common outcome measure is the modified Rankin Scale (mRS). However, the mRS is not especially sensitive to deficits that may improve most, such as aphasia. It is more sensitive to motor functions that impede walking. Many previous authors have reviewed the outcome measures used in acute stroke trials (e.g., 13, 14). One study recommended adding “extended/instrumental activities and advanced mobility as components of the primary outcome measure”^13^. Although the mRS was the common outcome assessment (64.3%); others used more sensitive measures such as the Barthel index (40.5%), but many failed to provide full details on outcome assessment methodology^14^. Some trials have addressed the limited sensitivity of the mRS by using the utility-weighted mRS (ranges from 0 [death] to 10 [no symptoms or disability])^15^. There are also ongoing collaborations among stroke trialists to develop more sensitive outcome measures especially for mild stroke, in order to evaluate the effectiveness of interventions for patients with mild deficits (e.g., mRS 0 or 1)^15^. It is hoped that future acute stroke trials will use a single, validated outcome measure that is sensitive to improvement (or deterioration) in all functions important to stroke patients.

There are important limitations to this study. We used items on the NIHSS as the only assessment of language, spatial attention, and strength. While the scoring is reliable, they are very limited measures of all of these functions. It is possible that fine motor control or other motor function might improve more than proximal strength measured by holding up each arm for 10 s and each leg for 5 s. Neglect/extinction was uncommon as measured with the NIHSS, and so we could not reliably compare percent improvement in this domain to other domains. A previous study showed that change in a more objective measurement of neglect (with simple line cancellation) actually correlated better with change in volume hypoperfusion than did change in the total NIHSS score in patients with right hemisphere stroke^12^. Furthermore, while we analyzed percentage change in NIHSS score as a continuous numerical value, the absolute change is a limited interval in scale. Since the NIHSS score questions for language and neglect are less granular (0–3 and 0–2 respectively) than for motor function (0–9 for each side of the body), it is possible that the NIHSS is more sensitive for motor function than for change in language or neglect (e.g., changing from moderate to mild aphasia is no change in score). However, the NIHSS also might over-estimate change in language. That is, a patient who improves from global aphasia to severe aphasia (or severe aphasia to mild-to-moderate aphasia) will have an improvement of 50% using our methodology. It may be more “difficult” to show an improvement of 50% in motor function. We did try to partially address this concern by evaluating change in “total language”, using points for correct answers to level of consciousness questions as well as the language item (for a range from 0 to 6), but the percentage change in language was not significantly different. The difference in scales for motor and language/neglect remains a limitation that we could not resolve. This study was also an observational study, retrospectively analyzing data from a prospectively collected sample of patients. Intervention was not randomized, but was generally determined by following American Heart Association guidelines for treatment of acute ischemic stroke. The aim of this study was not, however, to compare interventions, but to compare improvements across functions in individuals in each intervention group.

We also did not report additional demographic details other than age and sex (e.g., education, medications, co-morbidities), because the patients in each comparison (e.g., percent improvement in language versus percent improvement in motor function) were the identical patients. That is, all patients who had language and/or motor deficits were included in the primary analyses, and each patient would have been scored for percent change in language, percent change in total language, and percent change in motor function. It is unclear what variables (other than reperfusion) would differentially affect language versus motor functions in the same individuals.

Finally, our hypothesis that motor function will not improve as much as language/neglect was based on the assumption that deeper areas of the brain suffer irreversible ischemia faster than cortical areas. This assumption was based on previous studies of evolution of infarct in acute stroke that used MRI or Positron Emission Topography^1,16^. However, it is a limitation of this study that we did not have a second image in these participants to show that deeper areas were more likely to evolve to infarct than cortical areas. Future studies to confirm our hypothesis should include analysis of both an initial image and follow-up image of the infarct, at the same times as behavioral testing. Future studies should also evaluate the influence of age and other variables that might affect outcome.

Nevertheless, results provide novel and important information about the likelihood and estimated degree of improvement in gross measures of aphasia, neglect, and weakness in acute ischemic stroke, which may be useful in clinical decision-making. Moreover, results can help clinician provide patients and families a realistic idea of what is likely to improve the most with treatment. In patients with both aphasia and weakness pretreatment, language is likely to improve to a greater degree than weakness with thrombolysis or EVT.

## Electronic supplementary material

Below is the link to the electronic supplementary material.

Supplementary Material 1

## Data Availability

The data that support the findings of this study are not openly available due to reasons of sensitivity and are available from the corresponding author upon reasonable request. Data are located in controlled access data storage at Johns Hopkins University School of Medicine.
