# Supplementary material for: Relative improvement in language vs. motor functions with reperfusion therapies for large vessel occlusion
Source: Sci Rep. 2025 Feb 24;15:6683. doi: 10.1038/s41598-025-90871-x (PMC11850585; doi:10.1038/s41598-025-90871-x)
Supplement: Supplementary file 1 — Supplementary Material 1 [file 41598_2025_90871_MOESM1_ESM.docx]

**Supplemental Table 1. Percent Change in each Function Among those with Pre-Treatment Deficits, in the EVT only group, and the EVT + thrombolysis groups.**

|  | Mean |  | Standard Deviation |  | 95% Confidence Interval |
| --- | --- | --- | --- | --- | --- |
| **EVT (with thrombolysis) (N=10)** |  |  |  |  |  |
| Percent Change in Language | 40.0 |  | 40.9 |  | 10.7 - 62.5 |
| Percent Change in Motor | 24.2 |  | 19.1 |  | 10.6 – 37.9 |
| **EVT (without thrombolysis) (N=13)** |  |  |  |  |  |
| Percent Change in Language | 51.3 |  | 35.0 |  | 30.1 - 72.4 |
| Percent Change in Motor | 31.2 |  | 19.0 |  | 19.7 – 42.7 |
